# Design, synthesis and biological profile of heterocyclic benzimidazole analogues as prospective antimicrobial and antiproliferative agents

**DOI:** 10.1186/s13065-019-0567-x

**Published:** 2019-04-03

**Authors:** Sumit Tahlan, Sanjiv Kumar, Kalavathy Ramasamy, Siong Meng Lim, Syed Adnan Ali Shah, Vasudevan Mani, Ranjana Pathania, Balasubramanian Narasimhan

**Affiliations:** 1 Faculty of Pharmaceutical Sciences, Maharshi Dayanand University, Rohtak, 124001 India; 2 Faculty of Pharmacy, Universiti Teknologi MARA (UiTM), 42300 Bandar Puncak Alam, Selangor Malaysia; 3 Collaborative Drug Discovery Research (CDDR) Group, Pharmaceutical Life Sciences Community of Research, Universiti Teknologi MARA (UiTM), 40450 Shah Alam, Selangor Malaysia; 4 Atta-ur-Rahman Institute for Natural Products Discovery (AuRIns), Universiti Teknologi MARA (UiTM), Puncak Alam Campus, 42300 Bandar Puncak Alam, Selangor Malaysia; 5 Department of Pharmacology and Toxicology, College of Pharmacy, Qassim University, Buraidah, 51452 Kingdom of Saudi Arabia; 6 Department of Biotechnology, Indian Institute of Technology, Roorkee, 247667 India

**Keywords:** Antibacterial, Antifungal, Anticancer, 2-Mercaptobenzimidazole, SAR

## Abstract

**Background:**

Nitrogen containing heterocycles are widely used and investigated by pharmaceutical industry, as they are important in discovery and designing of new drug molecules. Drugs with a benzimidazole nucleus possess exclusive structural features and electron-rich atmosphere, which enable them to bind to a number of biologically important targets and result in a wide range of activities. This has served as the basis of the present study whereby new scaffolds with benzimidazole moiety were designed and synthesized.

**Methods:**

The structures of synthesized compounds were confirmed by physicochemical and spectral means. The synthesized compounds were screened for their antimicrobial and antiproliferative activities by tube dilution and Sulforhodamine B (SRB) assays, respectively.

**Results and conclusion:**

The in vitro biological screening results revealed that compound **Z24** exhibited promising antimicrobial and anticancer activities which are comparable to standards.
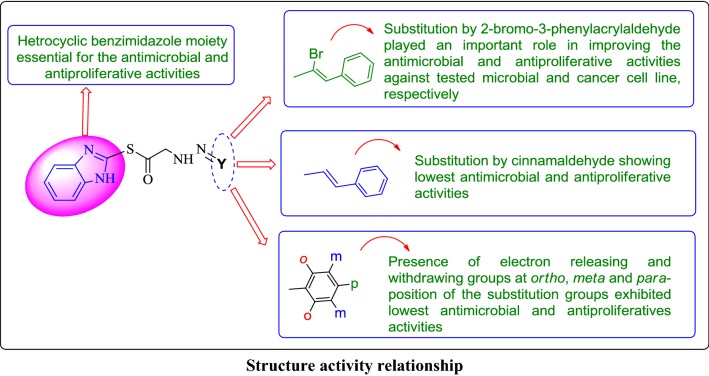

## Introduction

The increased incidences of drug resistance caused by extensive use of antibiotics and immunosuppressive drugs have emerged as a key issue in treatment of microbial infections. There is, therefore, a need for search of efficient, less toxic and structurally new molecules for treatment of these diseases. The discovery and development of drugs with benzimidazole moiety is now an important and attractive subject of interest given their huge therapeutic values [1]. Heterocyclic compounds offer a high degree of structural diversity and have proven to be broadly and economically useful as therapeutic agents like quinoline-branched amines and dimers [2], 8-substituted quinolines [3], 2,5- and 4,5-dihydroisoxazole [4].

The structural isosters of nucleotides, benzimidazole and heterocycles, which interact with biopolymer through the fused heterocyclic nuclei in their structure, may possess potential activity of chemotherapeutics with lower toxicity. It is well established that heterocyclic compounds with nitrogen and sulphur exhibit a wide scope of biological activities. 2-Mercaptobenzimidazole, for example, has been reported for their wide range of pharmacological and clinical applications [5]. On the other hand, azomethine group which is present in various natural and non-natural compounds, is also an important scaffold critical for biological activity of Schiff bases. Owing to their broad spectrum of biological profile, Schiff bases derived from benzimidazole compounds are extensively studied [6].

Colorectal cancer (CRC) is one of the most common malignancies and a noteworthy reason for growth related mortality around the world. According to World Health Organization (WHO), CRC is the third most regular malignancy, with 1,361,000 cases worldwide. Unfortunately, about 25% of CRC cases are only identified at stage IV (with far off metastases) and nearly half of CRC patients suffered from metastasis in the midst of their lifetime. The treatment outcomes for these patients remain inauspicious whilst approximately half of the patients responded to traditional chemotherapy, most encountered resistance at some stage of treatment, and reoccurrence of the tumors regularly take after. This could be due to cancer stem cells (CSCs) which give rise to heterogeneity within and between tumors [7].

**Fig. 1:**
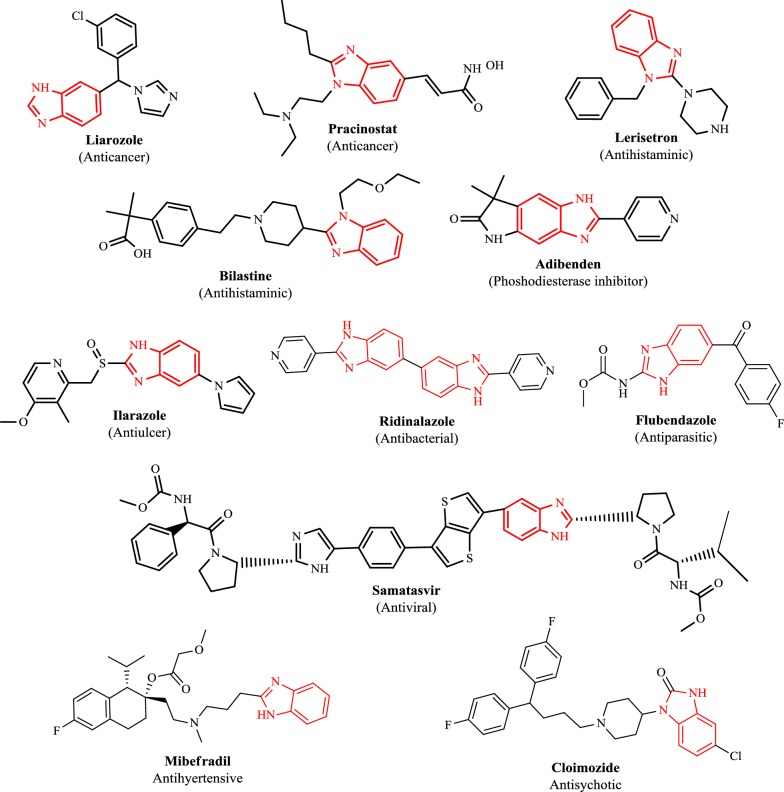
Marketed medicines containing benzimidazole as core moiety

In recent years, remarkable attention has been directed towards the advancement of benzimidazole heterocyclic molecules as antihistaminic (H_1_-receptor antagonist, e.g. bilastine, 5-HT_3_ antagonist, e.g. lerisetron) [8], antimicrobial (antibiotic, e.g. ridinilazole) [9, 10], antiulcer (proton pump inhibitor (PPI), e.g. ilaprazole) [11, 12], antihypertensive (calcium channel blocker, e.g. mibefradil) [13], antiviral (non-structural protein inhibitor (NS5A), e.g. samatasvir) [14, 15], antiparasitic (specifically anthelmintic, e.g. flubendazole) [16], antipsychotic (D_2_ receptor antagonist, e.g. clopimozide) [17], analgesic (opioid analgesic, e.g. clonitazene) [18], phosphodiesterase inhibitor (PDE3 inhibitor e.g. adibendan) [19, 20] and anticancer (aromatase inhibitor, e.g. liarozole, histone deacetylase inhibitor (HDAC), e.g. pracinostat) [21, 22] agents (Fig. 1).

**Fig. 2:**
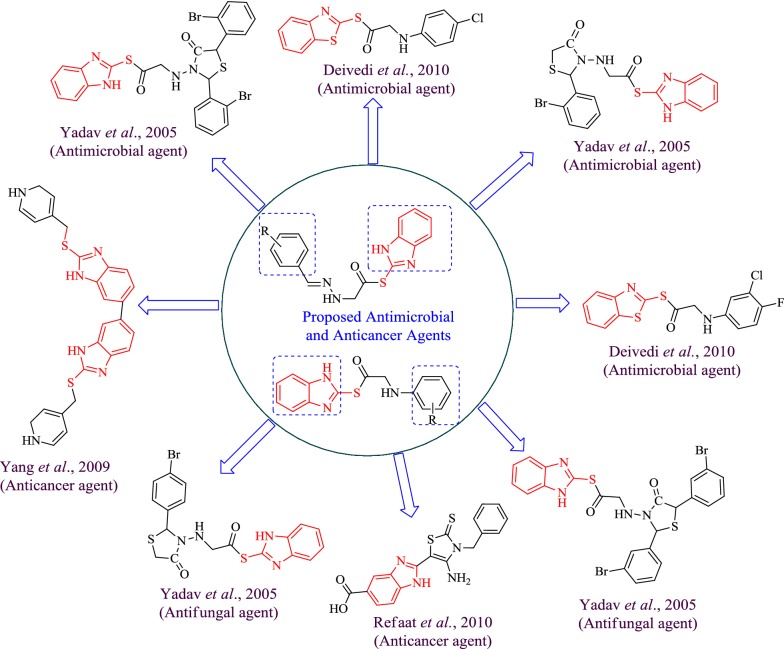
Design of benzimidazole analogues for antimicrobial and anticancer activity based on biological profile

Prompted by the aforementioned facts and literature on pharmacologically active heterocyclic benzimidazole nucleus (as reviewed in Fig. 2), the present work aimed to synthesize a new series of benzimidazole compounds and evaluate their biological activities.

## Results and discussion

### Chemistry

**Scheme 1:**
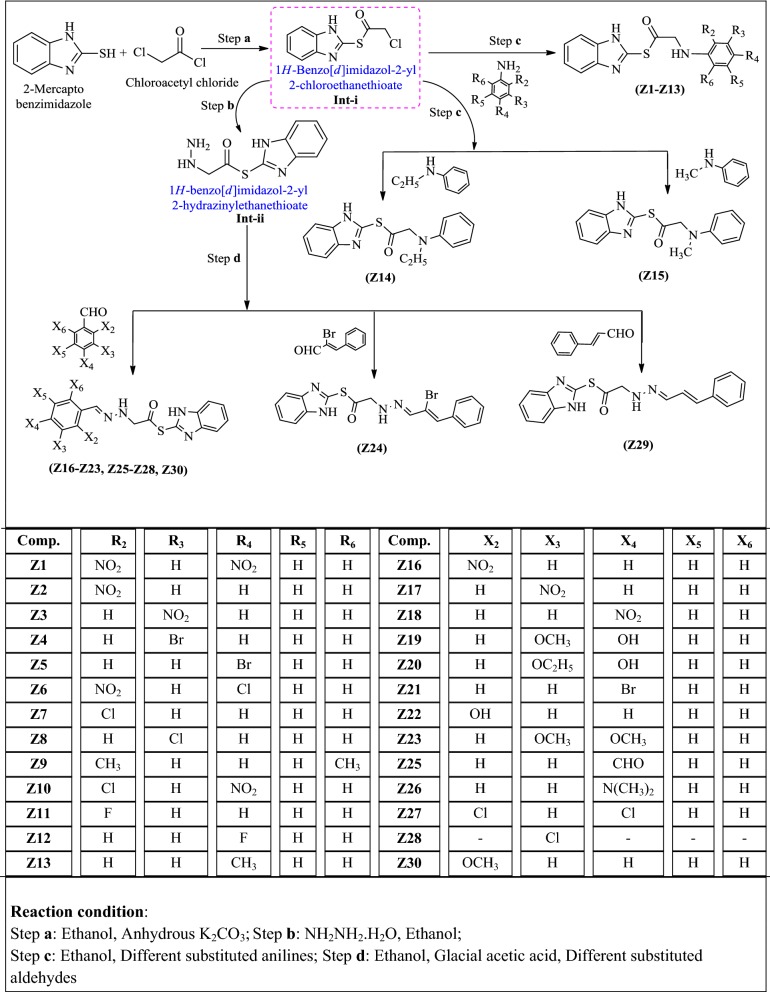
Synthesis of heterocyclic benzimidazole derivatives

The synthesis of benzimidazole derivatives (**Z1**–**Z30**) by multistep procedure was shown in Scheme 1. The 1*H*-benzo[*d*]imidazol-2-yl 2-chloroethanethioate (intermediate-**i**) was synthesized by the reaction of chloroacetyl chloride with 2-mercaptobenzimidazole, which on further reaction with corresponding anilines in presence of ethanolic solvent yielded the title compounds (**Z1**–**Z15**). The reaction of above synthesized intermediate-**i** with hydrazine hydrate yielded intermediate**-ii**. The intermediate**-ii** on reaction with substituted aldehydes in ethanol resulted in development of title compounds (**Z16**–**Z30**) with appreciable yields. The physicochemical properties of compounds (**Z1**–**Z30**) are shown in Table 1.

**Table 1:** The physicochemical properties of synthesized benzimidazole derivatives

Comp.	IUPAC name	Mol. structure	Mol. formula	R*f* value^a^	m. p. (^o^C)	% yield
**Z1**	*1H*-*Benzo[d]imidazol*-*2*-*yl 2*-*(2,4*-*dinitrophenyl*-*amino) ethanethioate*		C_15_H_11_N_5_O_5_S	0.48	180–183	83.64
**Z2**	*1H*-*Benzo[d]imidazol*-*2*-*yl 2*-*(2*-*nitrophenylamino)ethanethioate*		C_15_H_12_N_4_O_3_S	0.82	212–215	73.93
**Z3**	*1H*-*Benzo[d]imidazol*-*2*-*yl 2*-*(3*-*nitrophenylamino)ethanethioate*		C_15_H_12_N_4_O_3_S	0.75	217–220	85.74
**Z4**	*1H*-*Benzo[d]imidazol*-*2*-*yl 2*-*(3*-*bromophenylamino)ethanethioate*		C_15_H_12_N_3_OSBr	0.47	257–260	73.19
**Z5**	*1H*-*Benzo[d]imidazol*-*2*-*yl 2*-*(4*-*bromophenylamino)ethanethioate*		C_15_H_12_N_3_OSBr	0.65	260–263	57.48
**Z6**	*1H*-*Benzo[d]imidazol*-*2*-*yl 2*-*(4*-*chloro*-*2*-*nitrophenylamino) ethanethioate*		C_15_H_11_N_4_O_3_SCl	0.66	140–143	94.19
**Z7**	*1H*-*Benzo[d]imidazol*-*2*-*yl 2*-*(2*-*chlorophenylamino)ethanethioate*		C_15_H_12_N_3_OSCl	0.60	277–280	60.63
**Z8**	*1H*-*Benzo[d]imidazol*-*2*-*yl 2*-*(3*-*chlorophenylamino)ethanethioate*		C_15_H_12_N_3_OSCl	0.68	265–268	65.87
**Z9**	*1H*-*Benzo[d]imidazol*-*2*-*yl 2*-*(2,6*-*dimethyyphenylamino) ethanethioate*		C_17_H_17_N_3_OS	0.59	274–277	65.52
**Z10**	*1H*-*Benzo[d]imidazol*-*2*-*yl 2*-*(2*-*chloro*-*4*-*nitrophenylamino) ethanethioate*		C_15_H_11_N_4_O_3_SCl	0.74	180–183	91.45
**Z11**	*1H*-*Benzo[d]imidazol*-*2*-*yl 2*-*(2*-*florophenylamino)ethanethioate*		C_15_H_12_N_3_OSF	0.73	272–275	72.43
**Z12**	*1H*-*Benzo[d]imidazol*-*2*-*yl 2*-*(4*-*florophenylamino)ethanethioate*		C_15_H_12_N_3_OSF	0.55	270–273	68.60
**Z13**	*1H*-*Benzo[d]imidazol*-*2*-*yl 2*-*(4*-*methylphenylamino)ethanethioate*		C_16_H_15_N_3_OS	0.61	266–269	64.13
**Z14**	*1H*-*Benzo[d]imidazol*-*2*-*yl 2 (methyl (phenyl)amino)ethanethioate*		C_17_H_17_N_3_OS	0.70	245–248	53.40
**Z15**	*H*-*Benzo[d]imidazol*-*2*-*yl 2*-*(ethy l(phenyl)amino)ethanethioate*		C_16_H_15_N_3_OS	0.75	261–264	56.95
**Z16**	*1H*-*Benzo[d]imidazol*-*2*-*yl 2*-*(2*-*(2*-*nitrobenzylidene)hydrazinyl) ethanethioate*		C_16_H_13_N_5_O_3_S	0.58	258–260	59.40
**Z17**	*1H*-*Benzo[d]imidazol*-*2*-*yl 2*-*(2*-*(3*-*nitrobenzylidene)hydrazinyl) ethanethioate*		C_16_H_13_N_5_O_3_S	0.54	262–265	82.89
**Z18**	*1H*-*Benzo[d]imidazol*-*2*-*yl 2*-*(2*-*(4*-*nitrobenzylidene)hydrazinyl) ethanethioate*		C_16_H_13_N_5_O_3_S	0.59	250–253	65.04
**Z19**	*1H*-*Benzo[d]imidazol*-*2*-*yl 2*-*(2*-*(4*-*hydroxy*-*3*-*methoxybenzylidene) hydrazinyl)ethanethioate*		C_17_H_16_N_4_O_3_S	0.53	263–266	53.93
**Z20**	*1H*-*Benzo[d]imidazol*-*2*-*yl 2*-*(2*-*(3*-*ethoxy*-*4*-*hydoxybenzylidene) hydrazinyl)ethanethioate*		C_18_H_18_N_4_O_3_S	0.52	269–272	60.65
**Z21**	*1H*-*Benzo[d]imidazol*-*2*-*yl 2*-*(2*-*(4*-*bromobenzylidene)hydrazinyl) ethanethioate*		C_16_H_13_N_4_OSBr	0.50	277–280	65.52
**Z22**	*1H*-*Benzo[d]imidazol*-*2*-*yl 2*-*(2*-*(2*-*hydroxybenzylidene)hydrazinyl) ethanethioate*		C_16_H_14_N_4_O_2_S	0.57	259–262	63.60
**Z23**	*1H*-*Benzo[d]imidazol*-*2*-*yl 2*-*(2*-*(3,4*-*dimethoxybenzylidene) hydrazinyl)ethanethioate*		C_18_H_18_N_4_O_3_S	0.46	264–267	72.30
**Z24**	*1H*-*Benzo[d]imidazol*-*2*-*yl 2*-*(2*-*(2*-*bromo*-*3*-*phenylallylidene) hydrazinyl)ethanethioate*		C_18_H_15_N_4_OSBr	0.51	160–163	78.68
**Z25**	*1H*-*Benzo[d]imidazol*-*2*-*yl 2*-*(2*-*(4*-*(dimethylamino)benzylidene) hydrazinyl)ethanethioate*		C_17_H_14_N_4_O_2_S	0.57	250–253	72.58
**Z26**	*1H*-*Benzo[d]imidazol*-*2*-*yl 2*-*(2*-*(4*-*formylbenzylidene) hydrazinyl)ethanethioate*		C_18_H_19_N_5_OS	0.65	200–203	87.45
**Z27**	*1H*-*Benzo[d]imidazol*-*2*-*yl 2*-*(2*-*(2,4*-*dichlorobenzylidene) hydrazinyl) ethanethioate*		C_16_H_12_N_4_OSCl_2_	0.49	255–257	81.69
**Z28**	*1H*-*Benzo[d]imidazol*-*2*-*yl 2*-*(2*-*(2*-*chlorobenzylidene)hydrazinyl) ethanethioate*		C_16_H_13_N_4_OSCl	0.64	259–262	74.33
**Z29**	*1H*-*Benzo[d]imidazol*-*2*-*yl 2*-*(2*-*(3*-*phenylallylidene)hydrazinyl) ethanethioate*		C_18_H_16_N_4_OS	0.56	264–267	64.68
**Z30**	*1H*-*Benzo[d]imidazol*-*2*-*yl 2*-*(2*-*(2*-*methoxylbenzylidene) hydrazinyl)ethanethioate*		C_17_H_16_N_4_O_2_S	0.48	267–270	57.12

^a^TLC mobile phase-ethyl acetate

### Spectral characterization data

The assigned molecular scaffolds of the benzimidazole derivatives were authenticated by Infrared (IR), Nuclear Magnetic Resonance (NMR) (proton, carbon), Mass spectrometry (MS) and elemental analysis. The spectroanalytical data has been presented in Table 2. The IR spectra of compounds exhibited the characteristic secondary (–C=N–) absorption bands around 1345–1254 cm^−1^ while the tertiary (–C=N–) bands was observed at 1386–1337 cm^−1^. Appearance of IR stretching vibrations at 1600 and 1450 cm^−1^ in the spectra of compounds showed the presence of aromatic **–**C=C– and the peaks at 3112–3068 cm^−1^. The N–N peak in the spectra of synthesized derivatives was observed at 1168–1163 cm^−1^. The presence of ketonic stretching vibrations was observed at 1732–1698 cm^−1^. In the synthesized derivatives, the methylene group (–CH_2_–) showed the vibrations at 2935–2915 cm^−1^ and 2884–2865 cm^−1^. The compounds (**Z1**–**Z3**, **Z6**, **Z10** and **Z16**–**Z18**) showed the characteristic NO_2_ group stretching vibrations in the range of 1512–1484 cm^−1^. The compounds (**Z4**, **Z5**, **Z21** and **Z24**) exhibited the peaks of Br at 700–600 cm^−1^ while compounds (**Z6**–**Z8**, **Z10**, **Z27** and **Z28**) showed peaks in the range of 744–738 cm^−1^ and the fluorine group indicated its peak at 1012–1007 cm^−1^. The methyl group present in the compounds (**Z9**, **Z13** and **Z20**) showed the bands at 2948–2870 cm^−1^. The N–CH_3_ stretching band in the compounds (**Z14**, **Z15** and **Z26**) was observed at 2857–2843 cm^−1^. The phenolic group present in the compounds (**Z19**, **Z20** and **Z22**) showed its peaks in the range of 3648–3645 cm^−1^. The aldehydic group was confirmed by the appearance of absorption bands at 1728 cm^−1^ in the compound **Z25**. The presence of OCH_3_ in compounds (**Z19**, **Z20**, **Z23** and **Z30**) was observed at peaks in the range of 2839–2818 cm^−1^. The ^1^H-NMR spectra of the synthesized compounds have been recorded in dimethyl sulfoxide (DMSO) solvent. Multiplet signals at 6.62–8.66 δ ppm indicated the aromatic protons. The presence of singlet signal at 1.04–2.12 δ ppm indicated the presence of NH group while the singlet signal at 5.61–7.18 δ ppm confirmed the presence of NH group in imidazole ring. The appearance of singlet signals at 2.00–3.82 δ ppm indicated the presence of –CH_2_ in the compounds. The appearance of singlet signal at 1.21–3.63 δ ppm indicated the presence of CH_3_ in **Z9**, **Z13**–**Z15**, **Z20** and **Z26**. The singlet signal at 3.59–3.75 δ ppm confirmed the appearance of OCH_3_ group in the compounds (**Z19**, **Z23** and **Z30**) while the singlet signal at 1.21 δ ppm indicated the presence of OC_2_H-_5_ in compound **Z20**. ^13^C-NMR spectral analyses exhibit the appearance of the carbon atoms in synthesized molecular structures is shown in experimental section. The elemental analyses of compounds were around ± 0.3% of the theoretical results.

**Table 2:** Spectral data of synthesized benzimidazole derivatives

Comp.	IR (ATR cm^−1^)	^13^C-NMR (DMSO-*d6*, δ ppm)	^1^H-NMR (DMSO-*d6*, δ ppm)	C, H, N analyses calculated (found); MS ES + (ToF): *m/z*—[M^+^+1]
**Z1**	{3074 (C–H str.), 1461 (C=C str.) of phenyl nucleus (pn)}, 1337 (C=N str.), 1258 (C–N str.), 706 (C–S str.), 2840 (C–H str., –CH_2_–), 1512 (C–NO_2_ str.), 1698 (C=O str.)	109.33, 119.56, 122.12, 123.10, 128.46, 132.16, 134.99, 149.64, 168.06	7.03–8.66 (m, 7H, Ar–H), 7.03 (s, 1H, NH of imidazole), 3.51 (s, 2H, CH_2_), 2.01 (s, 1H, NH)	C, 48.26; H, 2.97; N, 18.76; (C, 48.22; H, 2.93; N, 18.72); 374
**Z2**	{3076 (C–H str.), 1459 (C=C str.) pn}, 1347 (C=N str.), 1254 (C–N str.), 695 (C–S str.), 2916 (C–H str., –CH_2_–), 1508 (C–NO_2_ str.), 1705 (C=O str.)	30.61, 109.41, 115.41, 119.11, 122.23, 125.31, 130.29, 132.20, 135.60, 146.12, 168.10, 206.31	6.62–7.99 (m, 8H, Ar–H), 6.62 (s, 1H, NH of imidazole), 3.49 (s, 2H, CH_2_), 2.10 (s, 1H, NH)	C, 54.87; H, 3.68; N, 17.06; (C, 54.83; H, 3.64; N, 17.02); 329
**Z3**	{3070 (C–H str.), 1457 (C=C str.) pn}, 1351 (C=N str.), 1334 (C–N str.), 696 (C–S str.), 2835 (C–H str., –CH_2_–), 1507 (C–NO_2_ str.), 1716 (C=O str.)	30.59, 107.01, 109.41, 109.75, 119.90, 122.23, 129.82, 132.20, 148.69, 150.01 168.10	6.99–7.44 (m, 8H, Ar–H), 6.99 (s, 1H, NH of imidazole), 3.49 (s, 2H, CH_2_), 2.10 (s, 1H, NH)	C, 54.87; H, 3.68; N, 17.06; (C, 54.91; H, 3.72; N, 17.10); 329
**Z4**	{3078 (C–H str.), 1461 (C=C str.) pn}, 1351 (C=N str.), 1333 (C–N str.), 698 (C–S str.), 2915 (C–H str., –CH_2_–), 654 (C–Br str.), 1719 (C=O str.)	109.40, 119.56, 122.22, 128.46, 132.16, 149.64, 168.06	6.82–7.11 (m, 8H, Ar–H), 6.82 (s, 1H, NH of imidazole), 3.63 (s, 2H, CH_2_), 1.05 (s, 1H, NH)	C, 49.73; H, 3.34; N, 11.60; (C, 49.77; H, 3.38; N, 11.64); 363
**Z5**	{3092 (C–H str.), 1462 (C=C str.) pn}, 1353 (C=N str.), 1335 (C–N str.), 701 (C–S str.), 2850 (C–H str., –CH_2_–), 656 (C–Br str.), 1714 (C=O str.)	109.40, 119.32, 122.22, 128.34, 132.18, 134.99, 149.64, 168.06	6.65–7.25 (m, 8H, Ar–H), 6.65 (s, 1H, NH of imidazole), 3.77 (s, 2H, CH_2_), 2.12 (s, 1H, NH)	C, 49.73; H, 3.34; N, 11.60; (C, 49.77; H, 3.38; N, 11.64); 363
**Z6**	{3083 (C–H str.), 1602 (C=C str.) pn}, 1356 (C=N str.), 1340 (C–N str.), 704 (C–S str.), 2850 (C–H str., –CH_2_–), 763 (C–Cl str.), 1503 (C–NO_2_ str.), 1716 (C=O str.)	109.37, 118.37, 121.02, 122.15, 123.94, 132.20, 135.41, 144.95, 168.10	7.06–7.22 (m, 8H, Ar–H), 7.06 (s, 1H, NH of imidazole), 3.51 (s, 2H, CH_2_), 2.12 (s, 1H, NH)	C, 49.66; H, 3.06; N, 15.44; (C, 49.70; H, 3.10; N, 15.48); 363
**Z7**	{3071 (C–H str.), 1454 (C=C str.) pn}, 1352 (C=N str.), 1334 (C–N str.), 691 (C–S str.), 2924 (C–H str., –CH_2_–), 739 (C–Cl str.), 1715 (C=O str.)	109.41, 119.65, 122.23, 128.56, 132.19, 134.99, 149.46, 168.09	7.16–7.22 (m, 8H, Ar–H), 7.16 (s, 1H, NH of imidazole), 3.61 (s, 2H, CH_2_), 2.12 (s, 1H, NH)	C, 56.69; H, 3.81; N, 13.22; (C, 56.65; H, 3.85; N, 13.18); 318
**Z8**	{3082 (C–H str.), 1459 (C=C str.) pn}, 1352 (C=N str.), 1334 (C–N str.), 695 (C–S str.), 2842 (C–H str., –CH_2_–), 738 (C–Cl str.), 1714 (C=O str)	109.40, 119.85, 122.21, 128.56, 132.18, 134.79, 149.46, 168.08	7.03–7.70 (m, 8H, Ar–H), 7.03 (s, 1H, NH of imidazole), 3.68 (s, 2H, CH_2_), 1.04 (s, 1H, NH)	C, 56.69; H, 3.81; N, 13.22; (C, 56.65; H, 3.77; N, 13.126); 318
**Z9**	{3091 (C–H str.), 1454 (C=C str.) pn}, 1352 (C=N str., N=CH), 1334 (C–N str.), 691 (C–S str., CH_2_–S), 2844 (C–H str., –CH_2_–), 2948 (C–H str., CH_3_), 1714 (C=O str., ketone)	30.59, 109.41, 119.56, 122.23, 128.41, 132.19, 134.52, 149.27, 168.10, 206.30	7.15–7.22 (m, 7H, Ar–H), 7.15 (s, 1H, NH of imidazole), 3.63 (s, 6H, (CH_3_)_2_), 2.11 (s, 1H, NH)	C, 65.57; H, 5.50; N, 13.49; (C, 65.53; H, 5.54; N, 13.45); 312
**Z10**	{3099 (C–H str.), 1623 (C=C str.) pn}, 1317 (C=N str.), 1305 (C–N str.), 704 (C–S str.), 2918 (C–H str., –CH_2_–), 744 (C–Cl str.), 1484 (C–NO_2_ str.), 1715 (C=O str.)	109.39, 113.46, 115.53, 122.18, 124.47, 125.51, 132.20, 135.92, 151.91, 168.10	6.91–7.99 (m, 7H, Ar–H), 6.91 (s, 1H, NH of imidazole), 3.59 (s, 2H, CH_2_), 2.12 (s, 1H, NH)	C, 49.66; H, 3.06; N, 15.44; (C, 49.62; H, 3.02; N, 15.40); 363
**Z11**	{3070 (C–H str.), 1454 (C=C str.) pn}, 1352 (C=N str.), 1335 (C–N str.), 693 (C–S str.), 2835 (C–H str., –CH_2_–), 1012 (C–F str.), 1716 (C=O str.)	30.60, 109.41, 115.21, 122.24, 132.19, 145.42, 150.32, 168.10;	7.14–7.20 (m, 8H, Ar–H), 7.14 (s, 1H, NH of imidazole), 3.53 (s, 2H, CH_2_), 2.10 (s, 1H, NH);	C, 59.79; H, 4.01; N, 13.94; (C, 59.75; H, 4.05; N, 13.90); 302
**Z12**	{3068 (C–H str.), 1454 (C=C str.) pn}, 1351 (C=N str.), 1332 (C–N str.), 691 (C–S str.), 2933 (C–H str., –CH_2_–), 1007 (C–F str.), 1714 (C=O str.)	109.40, 112.47, 122.23, 125.27, 132.19, 137.90, 146.32, 168.09	7.10–7.24 (m, 8H, Ar–H), 7.10 (s, 1H, NH of imidazole), 3.69 (s, 2H, CH_2_), 2.11 (s, 1H, NH)	C, 59.79; H, 4.01; N, 13.94; (C, 59.83; H, 4.04; N, 13.98); 302
**Z13**	{3073 (C–H str.), 1455 (C=C str.) pn}, 1351 (C=N str.), 1333 (C–N str.), 693 (C–S str.), 2844 (C–H str., –CH_2_–), 2870 (C–H str., CH_3_), 1713 (C=O str.)	30.62, 109.41, 114.34, 122.24, 125.86, 127.89, 132.20, 144.56, 149.64, 168.10	7.13–7.19 (m, 8H, Ar–H), 7.13 (s, 1H, NH of imidazole), 3.47 (s, 3H, CH_3_), 2.10 (s, 1H, NH)	C, 64.62; H, 5.08; N, 14.13; (C, 64.66; H, 5.04; N, 14.10); 298
**Z14**	{3069 (C–H str.), 1454 (C=C str.) pn}, 1352 (C=N str.), 1335 (C–N str.), 691 (C–S str.), 2914 (C–H str., –CH_2_–), 2850 (C–H str., N–CH_3_), 1714 (C=O str.)	30.96, 109.42, 114.37, 122.25, 128.79, 132.21, 145.64, 168.11	7.14–7.20 (m, 9H, Ar–H), 7.14 (s, 1H, NH of imidazole), 3.51 (q, 2H, –CH_2_), 2.10 (t, 3H, CH_3_)	C, 65.57; H, 5.50; N, 13.49; (C, 65.53; H, 5.54; N, 13.45); 312
**Z15**	{3079 (C–H str.), 1458 (C=C str.) pn}, 1344 (C=N str.), 1327 (C–N str.), 693 (C–S str.), 2844 (C–H str., –CH_2_–), 2857 (C–H str., N–CH_3_), 1706 (C=O str.)	30.59, 109.41, 114.33, 122.23, 127.89, 132.19, 139.27, 144.75, 168.10	7.08–7.22 (m, 9H, Ar–H), 7.08 (s, 1H, NH of imidazole), 3.61 (s, 31H, CH_2_), 2.11 (s, 3H, CH_3_)	C, 64.62; H, 5.08; N, 14.13; (C, 64.58; H, 5.12; N, 14.17); 298
**Z16**	{3105 (C–H str.), 1455 (C=C str.) pn}, 1701 (–CO str.), 1386 (C=N str.), 1343 (C–N str.), 1165 (N–N str., hydrazide), 694 (C–S str.), 2915 (C–H str., –CH_2_–), 1510 (C–NO_2_ str.)	109.41, 114.33, 119.17, 122.23, 126.32, 132.19, 137.42, 144.27, 168.09	7.04–7.35 (m, 8H, Ar–H), 7.04 (s, 1H, NH of imidazole), 2.11 (s, 1H, NH), 12.62 (s, 1H, N=CH)	C, 54.08; H, 3.69; N, 19.71; (C, 54.04; H, 3.65; N, 19.75); 356
**Z17**	{3112 (C–H str.), 1600 (C=C str.) pn}, 1701 (–CO str.), 1355 (C=N str.), 1337 (C–N str.), 1178 (N–N str., hydrazide), 702 (C–S str.), 2844 (C–H str., –CH_2_–), 1512 (C–NO_2_ str.)	109.39, 122.21, 130.92, 132.18, 139.87, 141.50, 145.71, 150.72, 168.08	7.18–8.26 (m, 8H, Ar–H), 6.84 (s, 1H, NH of imidazole), 3.55 (s, 2H, CH_2_), 2.12 (s, 1H, NH), 12.66 (s, 1H, N=CH)	C, 54.08; H, 3.69; N, 19.71; (C, 54.03; H, 3.73; N, 19.67); 356
**Z18**	{3106 (C–H str.), 1604 (C=C str.) pn}, 1703 (–CO str.), 1337 (C=N str.), 1270 (C–N str.), 1174 (N–N str., hydrazide), 697 (C–S str.), 2843 (C–H str., –CH_2_–), 1509 (C–NO_2_ str.)	61.89, 111.30, 122.91, 123.16, 130.29, 132.18, 139.78, 146.17, 146.54, 150.27, 191.68	7.23–8.38 (m, 8H, Ar–H), 5.61 (s, 1H, NH of imidazole), 3.57 (s, 2H, CH_2_), 2.12 (s, 1H, NH), 12.63 (s, 1H, N=CH)	C, 54.08; H, 3.69; N, 19.71; (C, 54.12; H, 3.64; N, 19.68); 356
**Z19**	{3081 (C–H str.), 1449 (C=C str.) pn}, 1716 (–CO str.), 1351 (C=N str.), 1331 (C–N str.), 1163 (N–N str., hydrazide), 689 (C–S str.), 2924 (C–H str., –CH_2_–), 3648 (O–H str.), 2839 (C–H str., –OCH_3_), 1256 (C–O–C str., phenyl ether)	30.60, 109.41, 111.19, 122.23, 127.89, 130.92, 132.19, 138.79, 145.92, 168.09	7.15–7.21 (m, 7H, Ar–H), 7.15 (s, 1H, NH of imidazole), 3.58 (s, 2H, CH_2_), 2.11 (s, 1H, NH), 12.60 (s, 1H, N=CH)	C, 57.29; H, 4.52; N, 15.72; (C, 57.25; H, 4.56; N, 15.76); 357
**Z20**	{3085 (C–H str.), 1454 (C=C str.) pn}, 1714 (–CO str.), 1352 (C=N str.), 1334 (C–N str.), 1166 (N–N str., hydrazide), 688 (C–S str.), 2852 (C–H str., –CH_2_–), 3645 (O–H str.), 2822 (C–H str., –OCH_3_), 1255 (C–O–C str., phenyl ether), 2901 (C–H str., CH_3_)	30.59, 109.41, 122.23, 125.21, 132.19, 130.18, 137.13, 144.65, 168.09	7.03–7.86 (m, 7H, Ar–H), 7.03 (s, 1H, NH of imidazole), 3.63 (s, 1H, OH), 2.11 (s, 1H, NH), 1.21 (t, 3H, CH_3_), 12.61 (s, 1H, N=CH)	C, 58.36; H, 4.90; N, 15.12; (C, 58.40; H, 4.94; N, 15.16); 371
**Z21**	{3109 (C–H str.), 1466 (C=C str.) pn}, phenyl nucleus), 1732 (–CO str.), 1356 (C=N str.), 1338 (C–N str.), 1178 (N–N str., hydrazide), 703 (C–S str.), 2842 (C–H str., –CH_2_–), 658 (C–Br str.)	30.59, 109.41, 122.23, 128.61, 131.05, 132.13, 132.19, 134.95, 192.01	7.17–7.22 (m, 8H, Ar–H), 7.17 (s, 1H, NH of imidazole), 3.66 (s, 2H, CH_2_), 2.11 (s, 1H, NH), 12.62 (s, 1H, N=CH)	C, 49.37; H, 3.37; N, 14.39; (C, 49.33; H, 3.33; N, 14.35); 390
**Z22**	{3091 (C–H str.), 1603 (C=C str.) pn}, 1703 (–CO str.), 1346 (C=N str.), 1294 (C–N str.), 1168 (N–N str., hydrazide), 690 (C–S str.), 2841 (C–H str., –CH_2_–), 3648 (O–H str.)	109.40, 122.22, 129.51, 131.03, 134.59, 146.32, 149.66, 168.08	7.18–7.24 (m, 8H, Ar–H), 7.18 (s, 1H, NH of imidazole), 3.74 (s, 1H, OH), 2.11 (s, 1H, NH), 12.64 (s, 1H, N=CH)	C, 58.88; H, 4.32; N, 17.17; (C, 58.84; H, 4.36; N, 17.13); 327
**Z23**	{3075 (C–H str.), 1452 (C=C str.) pn}, 1730 (–CO str.), 1380 (C=N str.), 1345 (C–N str.), 1170 (N–N str., hydrazide), 695 (C–S str.), 2876 (C–H str., –CH_2_–), 2835 (C–H str., –OCH_3_), 1253 (C–O–C str., phenyl ether)	109.40, 119.38, 122.22, 123.45, 128.67, 131.55, 132.18, 147.82, 168.08	7.18–7.24 (m, 7H, Ar–H), 7.18 (s, 1H, NH of imidazole), 375 (s, 6H, (OCH_3_)_2_), 2.12 (s, 1H, NH), 12.64 (s, 1H, N=CH)	C, 58.36; H, 4.90; N, 15.12; (C, 58.40; H, 4.94; N, 15.16); 371
**Z24**	{3087 (C–H str.), 1601 (C=C str.) pn}, 1713 (–CO str.), 1354 (C=N str.), 1337 (C–N str.), 1176 (N–N str., hydrazide), 694 (C–S str.), 2843 (C–H str., –CH_2_–), 684 (C–Br str.), 1623 (conjugated C=C and phenyl subst. C=C)	109.42, 122.21, 123.89, 128.66, 128.90, 130.55, 131.36, 132.23, 132.82, 150.14, 187.80	7.13–7.94 (m, 9H, Ar–H), 7.06 (s, 1H, NH of imidazole), 2.00 (s, 2H, CH_2_), 7.06 (s, 1H, NH), 12.55 (s, 1H, N=CH), 7.19 (s, 1H, Br–C=CH)	C, 52.06; H, 3.64; N, 13.49; (C, 52.02; H, 3.68; N, 13.45); 416
**Z25**	{3070 (C–H str.), 1453 (C=C str.) pn}, 1712 (–CO str.), 1352 (C=N str.), 1334 (C–N str.), 1167 (N–N str., hydrazide), 688 (C–S str.), 2846 (C–H str., –CH_2_–), 1728 (C–H str., CHO)	78.04, 111.42, 122.92, 129.61, 129.94, 135.88, 139.55, 145.74, 192.70	7.25–7.98 (m, 8H, Ar–H), 7.03 (s, 1H, NH of imidazole), 3.61 (s, 2H, CH_2_), 2.11 (s, 1H, NH), 12.60 (s, 1H, N=CH), 10.04 (s, 1H, CHO)	C, 60.34; H, 4.17; N, 16.56; (C, 60.38; H, 4.13; N, 16.52); 384
**Z26**	{3105 (C–H str.), 1453 (C=C str.) pn}, 1713 (–CO str.), 1352 (C=N str.), 1332 (C–N str.), 1163 (N–N str., hydrazide), 686 (C–S str.), 2927 (C–H str., –CH_2_–), 2843 (C–H str., N–CH_3_)	30.55, 39.49, 109.40, 110.89, 122.24, 124.45, 132.22, 154.02, 189.66	7.17–7.86 (m, 8H, Ar–H), 7.11 (s, 1H, NH of imidazole), 3.78 (s, 2H, CH_2_), 2.12 (s, 1H, NH), 12.65 (s, 1H, N=CH), 2.64 (s, 6H, (CH_3_)_2_)	C, 61.17; H, 5.42; N, 19.81; (C, 61.13; H, 5.46; N, 19.85); 354
**Z27**	{3092 (C–H str.), 1596 (C=C str.) pn}, 1710 (–CO str.), 1354 (C=N str.), 1336 (C–N str.), 1175 (N–N str., hydrazide), 700 (C–S str.), 2930 (C–H str., –CH_2_–), 739 (C–Cl str.)	61.91, 109.39, 122.20, 127.94, 130.66, 132.18, 133.65, 135.28, 137.04, 139.60, 188.40	6.74–7.72 (m, 7H, Ar–H), 6.74 (s, 1H, NH of imidazole), 3.02 (s, 2H, CH_2_), 2.11 (s, 1H, NH), 12.62 (s, 1H, N=CH)	C, 50.67; H, 3.19; N, 14.77; (C, 50.63; H, 3.15; N, 14.73); 380
**Z28**	{3111 (C–H str.), 1598 (C=C str.) pn}, 1703 (–CO str.), 1355 (C=N str.), 1337 (C–N str.), 1177 (N–N str., hydrazide), 701 (C–S str.), 2845 (C–H str., –CH_2_–), 740 (C–Cl str.)	30.58, 109.41, 119.56, 122.23, 124.54, 131.23, 132.18, 147.12, 168.08	7.16–7.28 (m, 8H, Ar–H), 7.16 (s, 1H, NH of imidazole), 3.65 (s, 2H, CH_2_), 2.11 (s, 1H, NH), 12.62 (s, 1H, N=CH)	C, 55.73; H, 3.80; N, 16.25; (C, 55.77; H, 3.84; N, 16.29); 345
**Z29**	{3110 (C–H str.), 1598 (C=C str.) pn}, 1712 (–CO str.), 1355 (C=N str.), 1337 (C–N str.), 1176 (N–N str., hydrazide), 700 (C–S str.), 2860 (C–H str., –CH_2_–), 1616 (phenyl conjugation)	109.40, 122.21, 128.72, 132.17, 134.54, 139.42, 141.72, 147.21, 168.07	7.18–7.27 (m, 9H, Ar–H), 7.18 (s, 1H, NH of imidazole), 3.82 (s, 2H, CH_2_), 2.12 (s, 1H, NH), 12.66 (s, 1H, N=CH)	C, 64.26; H, 4.79; N, 16.65; (C, 64.22; H, 4.75; N, 16.69); 337
**Z30**	{3103 (C–H str.), 1599 (C=C str.) pn}, 1713 (–CO str.), 1352 (C=N str.), 1335 (C–N str.), 1168 (N–N str., hydrazide), 691 (C–S str., CH_2_–S), 2841 (C–H str., –CH_2_–), 2818 (C–H str., –OCH_3_), 1255 (C–O–C str., phenyl ether)	109.41, 115.26, 122.23, 124.30, 132.19, 141.50, 145.71, 168.09, 205.61	7.15–7.25 (m, 8H, Ar–H), 7.15 (s, 1H, NH of imidazole), 3.70 (s, 2H, CH_2_), 2.10 (s, 1H, NH), 12.60 (s, 1H, N=CH), 3.59 (s, 1H, OH)	C, 59.98; H, 4.74; N, 16.46; (C, 59.94; H, 4.98; N, 16.42); 341

### Antimicrobial and anticancer screening results

**Fig. 3:**
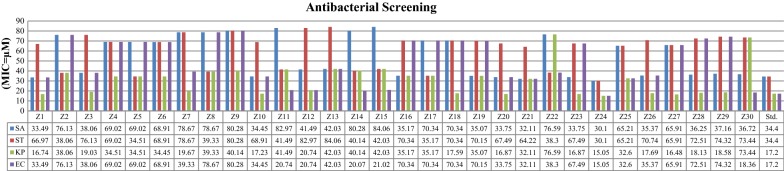
Antibacterial screening results against bacterial species

**Fig. 4:**
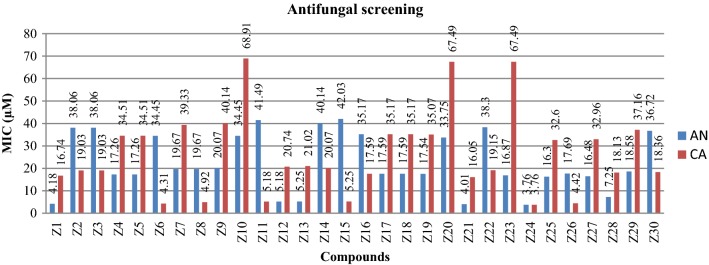
Antifungal screening results against fungal species

The synthesized benzimidazole compounds were screened for antimicrobial potential by tube dilution method using cefadroxil (antibacterial) and fluconazole (antifungal) as standard drugs against the represented microbial species. Furthermore, the anticancer activity of synthesized compounds against human colorectal carcinoma [HCT116 (ATCC CCL-247)] cancer cell line was assessed by using SRB assay with 5-fluorouracil (5-FU) being included as the standard anticancer drug. Biological screening results revealed that compound **Z24** displayed highest antimicrobial activity against Gram positive and Gram negative microorganisms (MIC_*sa, st*_ = 30.10 µM, MIC_*kp,ec*_ = 15.05 µM and MIC_*ca, an*_ = 3.76 µM). Besides, **Z24** also elicited anticancer activity against HCT116 cell line (IC_50_ = 0.46 µM) which was more potent than the standard drug. The antimicrobial screening results are presented in Table 3 and Figs. 3, 4 whereas the anticancer results are presented in Table 4.

**Table 3:** Antimicrobial screening results of synthesized derivatives

Comp.	Minimum inhibitory concentration (MIC = µM)					
	Bacterial strains				Fungal strains	
	*SA* MTCC3160	*ST* MTCC3231	*KP* MTCC9024	*EC* MTCC443	*AN* MTCC281	*CA* MTCC227
**Z1**	33.49	66.97	16.74	33.49	4.18	16.74
**Z2**	76.13	38.06	38.06	76.13	38.06	19.03
**Z3**	38.06	76.13	19.03	38.06	38.06	19.03
**Z4**	69.02	69.02	34.51	69.02	17.26	34.51
**Z5**	69.02	34.51	34.51	69.02	17.26	34.51
**Z6**	68.91	68.91	34.45	68.91	34.45	4.31
**Z7**	78.67	78.67	19.67	39.33	19.67	39.33
**Z8**	78.67	39.33	39.33	78.67	19.67	4.92
**Z9**	80.28	80.28	40.14	80.28	20.07	40.14
**Z10**	34.45	68.91	17.23	34.45	34.45	68.91
**Z11**	82.97	41.49	41.49	20.74	41.49	5.18
**Z12**	41.49	82.97	20.74	20.74	5.18	20.74
**Z13**	42.03	84.06	42.03	42.03	5.25	21.02
**Z14**	80.28	40.14	40.14	20.07	40.14	20.07
**Z15**	84.06	42.03	42.03	21.02	42.03	5.25
**Z16**	35.17	70.34	35.17	70.34	35.17	17.59
**Z17**	70.34	35.17	35.17	70.34	17.59	35.17
**Z18**	70.34	70.34	17.59	70.34	17.59	35.17
**Z19**	35.07	70.15	35.07	70.15	17.54	35.07
**Z20**	33.75	67.49	16.87	33.75	33.75	67.49
**Z21**	32.11	64.22	32.11	32.11	4.01	16.05
**Z22**	76.59	38.30	76.59	38.30	38.30	19.15
**Z23**	33.75	67.49	16.87	67.49	16.87	67.49
**Z24**	30.10	30.10	15.05	15.05	3.76	3.76
**Z25**	65.21	65.21	32.60	32.60	16.30	32.60
**Z26**	35.37	70.74	17.69	35.37	17.69	4.42
**Z27**	65.91	65.91	16.48	65.91	16.48	32.96
**Z28**	36.25	72.51	18.13	72.51	7.25	18.13
**Z29**	37.16	74.32	18.58	74.32	18.58	37.16
**Z30**	36.72	73.44	73.44	18.36	36.72	18.36
**DMSO**	NA	NA	NA	NA	NA	NA
**Broth control**	NG	NG	NG	NG	NG	NG
**Std.**	34.40^a^	34.40^a^	17.20^a^	17.20^a^	10.20^b^	10.20^b^

*SA*, *Staphylococcus aureus*; *ST*, *Salmonella typhi*; *KP*, *Klebsiella pneumoniae*; *EC*, *Escherichia coli*; *CA*, *Candida albicans*; *AN*, *Aspergillus niger*; DMSO, Dimethyl sulfoxide; NA, No activity; NG, No growth

Std drugs: ^a^Cefadroxil; ^b^Fluconazole

**Table 4:** Anticancer screening results of synthesized derivatives

Anticancer screening			
Comp.	IC_50_ (μM)	Comp.	IC_50_ (μM)
**Z1**	214.30	**Z16**	> 281.37
**Z2**	> 304.51	**Z17**	140.69
**Z3**	> 304.51	**Z18**	121.83
**Z4**	220.87	**Z19**	> 280.58
**Z5**	220.87	**Z20**	> 269.98
**Z6**	179.16	**Z21**	> 256.87
**Z7**	> 314.66	**Z22**	> 306.37
**Z8**	314.66	**Z23**	> 269.98
**Z9**	> 321.13	**Z24**	0.46
**Z10**	152.70	**Z25**	78.25
**Z11**	> 331.90	**Z26**	198.08
**Z12**	> 331.90	**Z27**	67.49
**Z13**	336.25	**Z28**	> 290.02
**Z14**	> 321.13	**Z29**	252.68
**Z15**	> 336.25	**Z30**	> 293.77
**5-Fluorouracil**	8.84	**5-Fluorouracil**	8.84

### Structure activity relationship (SAR)

**Fig. 5:**
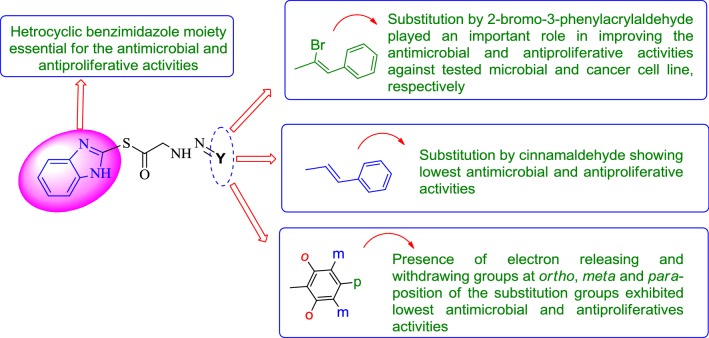
Structural requirements for the antimicrobial and anticancer activities of synthesized benzimidazole derivatives

The substitution of halogenated *α*, *β*-unsaturated aldehyde i.e. 2-bromo-3-phenylacrylaldehyde (compound **Z24**) displayed an important role in improving the antiproliferative and antimicrobial activities as compared to (compound **Z29**) its non-halogenated *α*, *β* unsaturated aldehyde. Structure activity relationship study is shown in Fig. 5.

## Methods/experimental

The starting material were purchased from different sources and used without further purification for the synthesis and analytical purpose. The reaction steps were confirmed by TLC (thin layer chromatography). Melting point was determined using labtech melting point equipment. An infrared spectrum was recorded [Attenuated total reflection (ATR), range of 4000–600 cm^−1^] on Bruker 12060280 spectrometer. Proton and carbon-NMR (*δ*, ppm) spectral analyses were determined by Bruker Avance III at 600 NMR and 150 MHz, respectively spectrometer in deuterated solvent. Waters Micromass Q-ToF Micro instrument was used for MS spectra. Elemental analyses were carried out by C, H and N analyzer (Perkin-Elmer 2400) around ± 0.3% of the theoretical results. The tested microorganism i.e. Gram positive, Gram negative and fungal species were procured from the Institute of Microbial Technology and Gene bank, Chandigarh for the in vitro antimicrobial activity.

### Procedure for synthesis of substituted benzimidazole derivative (**Z1**–**Z30**)

#### Step a: Synthesis of intermediate-**i**

A mixture of 2-mercaptobenzimidazole (0.01 mol) and chloroacetylchloride (0.01 mol) in ethanol (30 mL) in presence of anhydrous K_2_CO_3_ (0.01 mol) was refluxed for 6 h. The precipitated solid was filtered, evaporated to dryness under reduced pressure to get intermediate-**i** [5].

#### Step b: Synthesis of intermediate-**ii**

The solution of intermediate-**i** (0.01 mol) and hydrazine hydrate (0.02 mol) in ethanol (20 mL) was refluxed for 5 h. The solution was then poured in ice cold water and resulting solid was filtered, dried and recrystallized from ethanol [23].

#### Step c: Synthesis of title compounds **Z1**–**Z15**

An equimolar mixture of intermediate-**i** and substituted aniline in ethanol was refluxed for 4–5 h. After completion of reaction, it was poured into ice cold water and the precipitated title compound was filtered, dried and recrystallized from ethanol [24].

#### Step d: Synthesis of title compounds **Z16**–**Z30**

An equimolar mixture of intermediate-**ii** and substituted aromatic aldehydes with 2–3 drops of glacial acetic acid in ethanol (20 mL) was refluxed for 6 h. The resultant precipitate was filtered and recrystallized from ethanol to yield the required compound [23].

## Biological evaluation

### Antimicrobial evaluation (MIC)

The antimicrobial evaluation of the synthesized derivatives was carried out by tube dilution method [25] towards selected Gram positive, Gram negative and fungal microorganisms shown in Table 3. The screening results were compared with standard drugs i.e. cefadroxil and fluconazole.

#### The stock solutions (100 µg/mL)

The tested compounds and standard drugs were prepared in dimethylsulfoxide and further diluted up to six concentrations [26].

#### Broth media

Sabouraud dextrose broth used for antifungal activity and double strength nutrient broth used for antibacterial activity.

#### Incubation periods

The compounds were incubated at 25 ± 1 °C for 7 days (fungi—*A. niger*), at 37 ± 1 °C for 24 h (bacteria) and at 37 ± 1 °C for 48 h (fungi—*C. albicans*), respectively.

### Anticancer evaluation (IC_50_)

The antiproliferative activity was determined by SRB assay. Briefly, HCT116 was seeded onto the 96 well plate at 2500 cells/well. The cells were allowed to attach overnight before being exposed to the respective compounds (0.01–100 µg/mL) for 72 h. The highest concentration of each compound tested (100 µg/mL) contained only 0.1% DMSO (non-cytotoxic). SRB assay [27] was then performed whereby the cells were fixed using trichloroacetic acid for 30 min at 4 °C and stained with 0.4% (*w/v*) SRB mixed with 1% acetic acid for 15 min. After five washes with 1% acetic acid solution, the protein-bound dye was extracted with 10 mM tris base solution. Optical density was read at 570 nm and IC_50_ (i.e. concentration required to inhibit 50% of the cells) of each compound was determined. Anticancer results were presented as mean IC_50_ of at least triplicates.

## Conclusion

In this study, the synthesized benzimidazole scaffolds were authenticated by their consistent spectral data. The antimicrobial potential of synthesized compounds was assessed against different fungal and bacterial species, along with their anticancer activity against HCT116 cell line. The activity results indicated that the presence of *α*-bromo group in benzylidene portion (compound **24**) played an important role in improving the antimicrobial as well as antiproliferative activities and may be used as a lead for the discovery of new antimicrobial and anticancer agents.

## Abbreviations

CRC: Colorectal Cancer
HDAC: Histone Deacetylase Inhibitor
WHO: World Health Organization
IR: Infrared
NMR: Nuclear Magnetic Resonance
MS: Mass Spectrometry
TLC: Thin Layer Chromatography
ATR: Attenuated Total Reflection
DMSO: Dimethyl Sulfoxide
SRB: Sulforhodamine B
HCT116: Human colorectal carcinoma 116
MIC: Minimum Inhibitory Concentration
5-FU: 5-Fluorouracil
µM: micro mole
SAR: Structure Activity Relationship
MTCC: Microbial Type Culture Collection
*SA*: *Staphylococcus aureus*
*ST*: *Salmonella typhi*
*KP*: *Klebsiella pneumoniae*
*EC*: *Escherichia coli*
*CA*: *Candida albicans*
*AN*: *Aspergillus niger*
